# *Apiotrichum terrigenum* sp. nov., a soil-associated yeast found in both the UK and mainland Europe

**DOI:** 10.1099/ijsem.0.001467

**Published:** 2016-12-01

**Authors:** Stephen A. James, Christopher J. Bond, Rachael Stanley, Sreenivas R. Ravella, Gábor Péter, Dénes Dlauchy, Ian N. Roberts

**Affiliations:** ^1^​ National Collection of Yeast Cultures (NCYC), Institute of Food Research, Norwich Research Park, Colney, Norwich NR4 7UA, UK; ^2^​ Imaging and Microscopy Group (IMG), Institute of Food Research, Norwich Research Park, Colney, Norwich NR4 7UA, UK; ^3^​ Institute of Biological, Environmental and Rural Sciences, Aberystwyth University, Aberystwyth SY23 3EE, UK; ^4^​ National Collection of Agricultural and Industrial Microorganisms, Faculty of Food Science, Szent István University, Budapest, Hungary

**Keywords:** yeast, basidiomycota, *Apiotrichum*, biogas reactor, soil-associated, novel species

## Abstract

Five arthroconidium-producing yeast strains representing a novel *Trichosporon*-like species were independently isolated from the UK, Hungary and Norway. Two strains (Bio4^T^ and Bio21) were isolated from biogas reactors used for processing grass silage, with a third strain (S8) was isolated from soil collected at the same UK site. Two additional strains were isolated in mainland Europe, one from soil in Norway (NCAIM Y.02175) and the other from sewage in Hungary (NCAIM Y.02176). Sequence analyses of the D1/D2 domains of the LSU rRNA gene and internal transcribed spacer (ITS) region indicated that the novel species belongs to the recently reinstated genus *Apiotrichum* and is most closely related to *Apiotrichum scarabaeorum*, a beetle-associated species first found in South Africa. Despite having similar physiological characteristics, the two species can be readily distinguished from one another by ITS sequencing. The species name *Apiotrichum terrigenum* sp. nov. is proposed to accommodate these strains, with Bio4^T^ (=CBS 11373^T^=NCYC 3540^T^) designated as the type strain. The Mycobank deposit number is MB817431.

*Apiotrichum* is an anamorphic basidiomycetous yeast genus which was created to accommodate a single species, *Apiotrichum porosum*, whose original description (in German) was based upon a solitary strain isolated from the exudate of a yew tree (*Taxus baccata*) in Hamburg, Germany (Stautz, 1931). This species was later transferred to the genus *Trichosporon* Behrend and renamed as *Trichosporon porosum*. This taxonomic reassignment came about as a result of the molecular and phenotypic characterization of three additional strains representing the genus *Trichosporon* isolated from soils collected in The Netherlands (Middelhoven *et al.*, 2001). Although the soil isolates differed morphologically from the *A. porosum* type strain (CBS 2040^T^) in their ability to produce fragmented mycelia (arthroconidia), rDNA sequencing revealed all four strains to have identical LSU D1/D2 sequences and so they were accepted to be conspecific. Furthermore, a phylogenetic analysis based on LSU D1/D2 sequences showed the strains to be most closely related to *Trichosporon* *sporotrichoides* and to clearly belong to the genus *Trichosporon* (see Fig. 2 of Middelhoven *et al.*, 2001).

However, the genus *Apiotrichum* was recently revived by Liu *et al.* (2015b), and its description emended, to accommodate species previously assigned to the brassicae/gracile and porosum clades of the genus *Trichosporon* (Middelhoven *et al.*, 2004; Sugita, 2011). This revival resulted from a major taxonomic revision of the class *Tremellomycetes* based on phylogenetic analyses of a multi-gene dataset which included the majority of the tremellomycetous yeasts as well as closely related filamentous taxa (Liu *et al.*, 2015a, b). Yeasts of the genus *Apiotrichum* are distributed widely throughout nature and include a number of soil-associated species such as *Apiotrichum* *dulcitum*, *A. laibachii* and *A. loubieri* (Sugita, 2011). *Apiotrichum domesticum* and *A. montevideense* are also of clinical importance as they are causative agents of human summer-type hypersensitivity pneumonitis (SHP), an allergic disease caused by the repeated inhalation of arthroconidia, which are often found in the houses of patients suffering from this disease. The genus currently contains 20 species, with *A. porosum* designated as the type species (Liu *et al.*, 2015b).

In the course of a study to catalogue and characterize the microbial communities present in a set of UK-based biogas reactors, three arthroconidium-producing yeasts were recovered, two from two of the reactors and a third from soil sampled at the same site. Two additional strains were subsequently identified on the basis of LSU D1/D2 sequencing. These were recovered in two separate studies, one from soil collected in Norway and the other from sewage collected in Hungary. In this paper, we propose *Apiotrichum terrigenum* sp. nov. to accommodate these strains.

The three UK strains were all isolated at the North Wyke Research Institute at Okehampton, Devon. Two strains, Bio4^T^ (=NCYC 3540^T^) and Bio21, were isolated from separate samples taken directly from identical laboratory-scale biogas reactors used for processing grass silage feedstock as well as other plant biomass, while a third strain (S8) was isolated from a soil sample collected on site. Two additional strains, NCAIM Y.02175 and NCAIM Y.02176, were isolated in separate studies carried out in mainland Europe. Strain NCAIM Y.02175 was isolated from soil collected in Trondheim (Norway), while strain NCAIM Y.02176 was isolated from sewage sludge collected in Budapest (Hungary). All strains were characterized biochemically, morphologically and physiologically according to the standard methods described by Kurtzman *et al.* (2011). The temperature for growth was determined by cultivation on YM (yeast extract-malt extract; 0.3 % yeast extract, 0.3 % malt extract, 0.5 % peptone, 1 % glucose; Difco) agar. Mating experiments were performed on corn meal agar, Gorodkowa agar, potassium acetate agar and YM agar, and plates were incubated at 20 and 25 °C for 1 month in pure and mixed cultures.

The variable D1/D2 domains of the LSU rRNA gene and ribosomal internal transcribed spacer (ITS) region were amplified by PCR directly from whole yeast cell suspensions as described previously by James *et al.* (1996). The LSU D1/D2 domain was amplified and sequenced using primers NL1 and NL4 (O’Donnell, 1993). The ITS region was amplified using primers ITS4 and ITS5, and sequenced using these primers as well as internal primers ITS2 and ITS3 (White *et al.*, 1990). The amplified DNA was checked by 1.0 % agarose gel electrophoresis, purified and concentrated using QIAquick PCR purification spin columns (Qiagen). A NanoDrop 1000 spectrophotometer (Thermo Scientific) was used for measuring DNA concentration, and samples were sequenced by commercial sequencing facilities (Eurofins MWG Operon, Germany and Biomi, Hungary). Sequence traces were edited manually, and consensus sequences were generated using the program seqman, version 7 (dnastar). The LSU D1/D2 sequences were compared pairwise using a fasta similarity search (Pearson & Lipman, 1988) and were aligned with the sequences of closely related taxa, retrieved from the EMBL sequence database, using the multiple alignment program clustal w (Thompson *et al.*, 1994) included in the mega version 6 software package (Tamura *et al.*, 2013). A phylogenetic tree was reconstructed using the neighbour-joining (NJ) program included in mega, with the Kimura 2-parameter (K2P) distance measure and *Apiotrichum porosum*, the type species of the genus *Apiotrichum* selected as the outgroup. Bootstrap support for the NJ tree was determined from 1000 replicates.

On the basis of LSU D1/D2 sequence analysis, the five arthroconidium-producing strains were all identified as belonging to the genus *Apiotrichum*, a basidiomycetous genus recently revived by Liu *et al.* (2015b) and comprising species previously classified as members of the genus *Trichosporon* and assigned to the brassicae/gracile and porosum clades (Middelhoven *et al.*, 2004; Sugita, 2011; Liu *et al.*, 2015a). Despite being isolated from different substrates (biogas reactor feedstock, sewage and soil) and collected independently at three separate sites in the UK and mainland Europe, all five strains were found to have identical LSU D1/D2 and ITS sequences, indicating they were conspecific and belonged to the same species.

A fasta sequence similarity search of the EMBL fungal sequence database revealed no other yeast taxon with a LSU D1/D2 sequence identical to these strains. In terms of pairwise sequence similarity, the closest taxa were three as yet undescribed isolates collected in Taiwan; namely *Trichosporon* sp. EN31S13 (99.7 %; 2 nt substitutions in 571 nt), *Trichosporon* sp. ES24S05 (99.5 %; 3 nt substitutions in 571 nt) and *Trichosporon* sp. SU16S02 (99.3 %; 4 nt substitutions in 571 nt). These isolates were all originally deposited as ‘*Trichosporon* sp.’ and so remain to be reclassified as strains of the genus *Apiotrichum* following the recent work by Liu *et al.* (2015a, b). However, it is pertinent to note that *Trichosporon* sp. ES24S05 has a LSU D1/D2 sequence identical to CBS 5601^T^, the type strain of *Apiotrichum* *scarabaeorum* (formerly *Trichosporon scarabaeorum*), and so may represent a Taiwanese isolate of this species. Within the genus *Apiotrichum*, *A. scarabaeorum* was the most closely related known (i.e. formally described) species, displaying 0.8 % sequence divergence (5 nt substitutions in 615 nt). Although somewhat limited, these levels of sequence divergence are still comparable to those observed between other members of the genus. For example, the species pair of *A. domesticum* and *A. montevideense* display 99.7 % identity (2 nt substitutions in 626 nt; Fig. 1), as does *Apiotrichum* *gamsii* and its close relative *Apiotrichum* *wieringae* (3 nt substitutions in 624 nt) (Middelhoven *et al.*, 2004). Indeed, in their seminal study on the biodiversity and systematics of basidiomycetous yeasts, Fell *et al.* (2000) observed that strains differing by two or more nucleotides in the LSU D1/D2 domain represented distinct taxa, whereas strains belonging to the same species had identical LSU D1/D2 sequences. A phylogenetic analysis based on LSU D1/D2 sequences showed the novel taxon, as represented by strain NCYC 3540^T^ (=Bio4^T^), to be related to species of the genus *Apiotrichum* previously assigned to the *Trichosporon* brassicae clade (Middelhoven *et al.*, 2004; Sugita, 2011). In fact, the novel taxon along with *A. scarabaeorum* and the three Taiwanese isolates (EN31S13, ES24S05 and SU16S02) form a distinct subclade (bootstrap value, 98 %) within the genus *Apiotrichum* (Fig. 1).

**Fig. 1.**
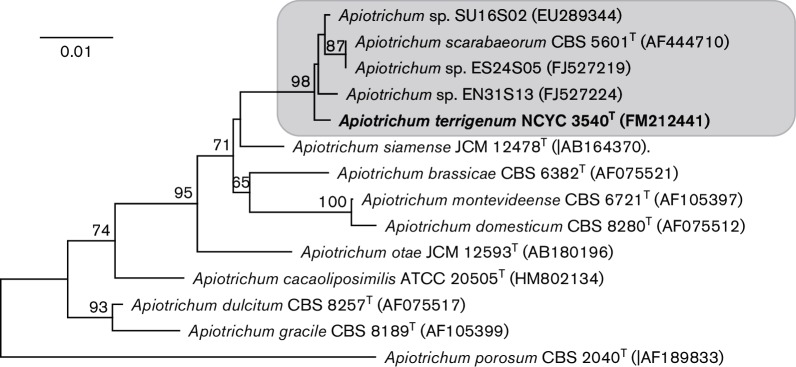
Neighbour-joining dendrogram based on sequences of the D1/D2 domain of the LSU rRNA gene of *Apiotrichum terrigenum* sp. nov. and its closest relatives. *A. porosum* was used as the outgroup species for the analysis. Bootstrap values of ≥50 %, determined from 1000 replicates, are shown at branch nodes. Bar, 1 base substitution per 100 nt.

Despite the close phylogenetic relationship based on LSU D1/D2 sequences (Fig. 1), the novel taxon and *A. scarabaeorum* can be readily distinguished from one another by ITS sequencing. In the ITS1 region, the two taxa differ by 10 nt substitutions and 2 indels (in 116 nt), and in the ITS2 region by 2 nt substitutions and 2 indels (in 173 nt). Overall, the two taxa display over 5 % sequence divergence in the entire ITS region (excluding the 5.8S rDNA). Indeed, with the exception *A. domesticum* and *A. montevideense*, whose respective ITS sequences differ by only a single nt indel (in the ITS1 region), all other species previously assigned to the *Trichosporon* brassicae clade can be readily distinguished from one another based on ITS sequencing (Fig. S1, available in the online Supplementary Material). Furthermore, and somewhat unexpectedly, two additional polymorphic sites were detected in the intervening and highly conserved 5.8S rDNA (Fig. S1). The first, a C→T transition (position 8; 5.8S rDNA numbering) was found in both *Apiotrichum* *brassicae* and *Apiotrichum* *otae*, while the second, an A→T transversion (position 54), was found in the novel taxon and *A. scarabaeorum*. Collectively, these two 5.8S rDNA single nucleotide polymorphisms (SNPs) can be used to subdivide the species of the genus *Apiotrichum* formerly assigned to the (*Trichosporon*) brassicae clade into three separate groups; the first comprising the novel taxon and *A.* *scarabaeorum*, the second *A. domesticum*, *A. montevideense* and *Apiotrichum* *siamense*, and the third *A. brassicae* and *A. otae* (Fig. S1).

From an ecological perspective, the novel species appears to be widely distributed and to date has been found in the UK, Hungary and Norway. Two strains were found in soil, one sample collected in the UK and the other in Norway. Two additional strains were isolated from separate biogas reactors (UK), while a fifth was recovered from sewage sludge (Hungary). Given the small sample size, it is difficult to determine the principal ecological niche of this species, although it is clearly present in soil, and a possible plant-association cannot be discounted, especially as it was recovered from biogas reactors used principally for processing grass silage. *A. scarabaeorum* currently represents its closest relative (Fig. 1) and, so far, this species has been isolated from the gut of a larval scarab beetle (South Africa; CBS 5601^T^), the rumen of a cow (New Zealand; CBS 4375) and also from a biogas reactor (UK; NCYC 3734). In fact, the latter strain was isolated in the same study, and from the same set of biogas reactors, as strains Bio4^T^ (=NCYC 3540^T^) and Bio21 (=NCYC 3737). This suggests the two closely related species of the genus *Apiotrichum* might inhabit a similar ecological niche. Soil certainly appears to be a substrate in which some members of the genus are commonly found, and these include *A. dulcitum* (Germany and The Netherlands), *A. gamsii* (Colombia), *A. laibachii* (Japan) and *A. vadense* (The Netherlands) (Sugita, 2011). Furthermore, *A. domesticum* and *A. loubieri* have even been found in soils collected from the same extreme habitat, namely the Dry Valleys in Antarctica, a large ice-free region dominated by low temperatures (annual average of −20 °C) and a lack of water (<10 cm precipitation year^−1^) (Fell *et al.*, 2006).

Physiologically, the novel species and *A. scarabaeorum* are almost indistinguishable from one another. The only standard growth characteristic that appears to be discriminatory is the assimilation of xylose. All three currently characterized strains of *A. scarabaeorum* (i.e. CBS 4375, CBS 5601^T^ and NCYC 3734) are able to assimilate this pentose sugar (this study; Sugita, 2011), whereas the novel species cannot. In fact, amongst the species of the genus *Apiotrichum* previously assigned to the *Trichosporon* brassicae clade (Middelhoven *et al.*, 2004; Sugita, 2011), only the novel species is unable to utilize xylose as a sole carbon source. With regard to the novel species, the assimilation of citrate, galactose, glycerol, soluble starch and sucrose all appear to be variable growth characteristics, allowing the five strains to be partially subdivided according to their differing geographical origins (i.e. UK or mainland Europe). The three UK strains can be distinguished from the Hungarian and Norwegian strains by their ability to assimilate sucrose (positive or slow). Likewise, the two strains from mainland Europe can both assimilate soluble starch (albeit slowly), whereas all three UK strains are unable to use this carbon source. Key growth characteristics that can be used to distinguish between the five novel strains as well as *A. scarabaeorum* are presented in Table S1. As yet, no phenotypic data is available for the three Taiwanese strains (EN31S13, ES24S05 and SU16S02).

In view of the fact these two species of the genus *Apiotrichum* have such similar overall phenotypic profiles, making accurate discrimination difficult, we strongly recommend that ITS sequencing is used as a far more reliable method for determining species identity. No intra-specific ITS sequence variation was detected between any of the five novel strains, irrespective of their origin (i.e. UK, Hungary or Norway). Likewise, no sequence variation was detected between the ITS sequences of *A. scarabaeorum* NCYC 3734 and the type strain (CBS 5601^T^). Again, this is despite the fact the two strains were isolated so far apart, one in the Northern Hemisphere (NCYC 3734, UK) and the other in the Southern Hemisphere (CBS 5601^T^, South Africa). Collectively, these data would suggest the ITS sequences of these species of the genus *Apiotrichum* are highly conserved and exhibit very limited, if any, intra-specific variation. This supports our proposal that the five novel strains clearly belong to a distinct species, rather than represent European variants of *A. scarabaeorum*.

## Description of *Apiotrichum terrigenum* James, Bond, Stanley, Péter and Roberts sp. nov.

*Apiotrichum terrigenum* (ter.ri′ge.num. L. masc. adj. *terrigenum*, born of the earth, earth-born).

**Fig. 2.**
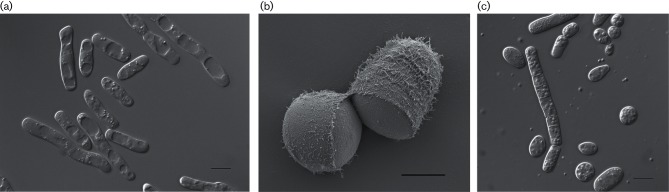
*Apiotrichum terrigenun* sp. nov. NCYC 3540^T^. (a) Photomicrograph of yeast cells in YM broth after 2 days at 25 °C. Bar, 10 µm. (b) Scanning electron microscope image of yeast cells grown with agitation in YM broth after 2 days at 25 °C. Bar, 5 µm. (c) Photomicrograph of septate hyphae and arthroconidia produced on YM agar after 7 days at 25 °C. Bar, 10 µm.

On YM agar, after 3 days of incubation at 25 °C, colonies are whitish to beige, dull and hirsute, often raised with a fimbriate margin. No extracellular polysaccharides are produced by any of the strains. In YM broth, after 2 days of incubation at 25 °C, cells are ovoid or elongate (6–10×9–51 µm) and occur singly, in pairs and in groups (Fig. 2a). Furthermore, scanning electron microscopy reveals that cultures of Bio4^T^ when grown in YM broth for 2 days at 25 °C exhibit a very interesting and distinct phenotypic characteristic, namely, a fibrillar-like cell surface (Fig. 2b). After 1 month, a pellicle (either creeping or static), and sediment are formed in the same medium. Septate hyphae with arthroconidia are produced on YM agar (Fig. 2c). No sexual reproduction has been observed.

Glucose is not fermented. Glucose, sucrose (variable), galactose (slow or negative), lactose (slow or weak), trehalose (slow, weak or negative), soluble starch (slow or negative), cellobiose (slow or weak), d-ribose (variable), ethanol (positive or slow), glycerol (variable), ribitol (weak or negative), d-mannitol (slow or weak), glucitol (variable), dl-lactate, succinate (positive or slow) and citrate (variable) are assimilated. No growth occurs on inulin, raffinose, melibiose, maltose, melezitose, salicin, l-sorbose, l-rhamnose, d-xylose, d-arabinose, l-arabinose, methanol, erythritol, galactitol, inositol, d-glucosamine or xylitol. Ethylamine hydrochloride (positive or slow), l-lysine (positive or slow) and cadaverine (variable) are assimilated, but not nitrate. Growth occurs at 30 °C, but not at 37 °C. Growth is variable on YM agar with 10 % (w/v) NaCl, but no growth occurs on either 50 % glucose/yeast extract or with 10 µg cycloheximide ml^−1^. Starch-like compounds are not produced. All strains are lipolytic whereas urease activity is variable.

The type strain, Bio4^T^ (=CBS 11373^T^=NCYC 3540^T^), was isolated from a grass silage feedstock sample collected directly from a laboratory-scale biogas reactor at the North Wyke Research Institute at Okehampton, Devon, UK. The Mycobank deposit number is MB817431.

## Supplementary Data

202 Supplementary File 1
