# Supplementary material for: Apiotrichum terrigenum sp. nov., a soil-associated yeast found in both the UK and mainland Europe
Source: Int J Syst Evol Microbiol. 2016 Dec 1;66(12):5046–50. doi: 10.1099/ijsem.0.001467 (PMC5244499; doi:10.1099/ijsem.0.001467)
Supplement: Supplementary File 1 [file ijsem-66-5046-s001.pdf]

Supplementary Data

Title: *Apiotrichum terrigenum* sp. nov., a novel soil-associated yeast found in both the UK and mainland Europe.

Authors: Stephen A. James, Christopher J. Bond, Rachael Stanley, Sreenivas R. Ravella, Gábor Péter, Dénes Dlauchy and Ian N. Roberts

Journal: International Journal of Systematic and Evolutionary Microbiology

Corresponding Author: Stephen A. James

Affiliation: National Collection of Yeast Cultures (NCYC), Institute of Food Research, Norwich Research Park, Colney, Norwich, UK.

email: steve.james@ifr.ac.uk

Fig. S1. ITS sequence alignment of *Apiotrichum terrigenum* sp. nov. Bio4^T (NCYC 3540^T) and other *Apiotrichum* species previously assigned to the *Trichosporon* brassicae clade. SSU (3' end), 5.8S (complete) and LSU (5' end) rDNA sequence is highlighted (in grey).

```
Bio4      AGGATCATTAGTGATTGCCTTAATTGGCT---AACTATATCC-ATCAACACCTGTGAACT     56
Ascar     .............................---..........-...T............A     56
Adome     .............................TA-..........-...T.............     58
Amont     .............................TA-..........-...T.............     58
Asiam     .....................C.......TAT..........-...T............C     59
Abras     .............................TA-..........-.A.T.............     58
Aotae     ...................A.C-......TA-..........C.................     48

Bio4      GTTCGATTGAATC--TCTTGGTTCAATTTT-TACAAACATTGTGTAATGAACGTCATT-T    112
Ascar     .............--.TAC.A.........--..........................AG    112
Adome     ...T.........--.TCG.A...G.....A...........................A.    116
Amont     ...T.........--.TCG.A...G.....A...........................A.    116
Asiam     ...T..C......CG.TAG.A..T.G....--..........................AG    117
Abras     .............--.T-C.A.........--...............A..........AG    113
Aotae     ..CT.........TT.A...A...G.....--.........................AAC    106

Bio4      ATTATAACATAAT-AAAACTTTCAACAACGGATCTCTTGGCTCTCGCATCGATGAAGAAC    171
Ascar     ..C.......G.A-..............................................    171
Adome     .........A..AA..............................................    176
Amont     .........A..A-..............................................    175
Asiam     ..C......G..A-..............................................    176
Abras     ..C......A..A-........T.....................................    172
Aotae     .......T.A..A-........T.....................................    165

Bio4      GCAGCGAATTGCGATAAGTAATGTGAATTGCAGAATTCAGTGAATCATCGAATCTTTGAA    231
Ascar     ............................................................    231
Adome     ........A...................................................    236
Amont     ........A...................................................    235
Asiam     ........A...................................................    236
Abras     ........A...................................................    232
Aotae     ........A...................................................    225

Bio4      CGCAACTTGCGCTCTCTGGTATTCCGGAGAGCATGCCTGTTTGAGTGTCATGAAATCTCA    291
Ascar     ............................................................    291
Adome     ............................................................    296
Amont     ............................................................    295
Asiam     ............................................................    296
Abras     ............................................................    292
Aotae     ............................................................    285

Bio4      ACCATTAGGGTTTCTTAATGGCTTGGATTTGGAGGTTTGCCAGTCTGACTGGCTCCTCTT    351
Ascar     .....................................-......................    350
Adome     ............................................................    356
Amont     ............................................................    355
Asiam     .....................................-......................    355
Abras     CA...C.A........G...AAG..............-........A.............    351
Aotae     .................................C...-.G....T.-....C...G....    343
```

```
Bio4      AAAGGAGTTAGCAAGTTTAACTATTGCTATCTGGCGTAATAAGTTTCGCTGGAATGGTAT    411
Ascar     ..................G.........................................    410
Adome     ...A.............G..........................................    416
Amont     ...A.............G..........................................    415
Asiam     ..................G............GA.................C-.G.A....    414
Abras     .............TA...G.T.......G.....................A.TT...C..    411
Aotae     ...A.............-..........G.........................A..C..    402

Bio4      TGTGAAGCGTGCTTCTAATCGTCTTCGGACAATTACTTT-GACTCTGGCCTCAA          464
Ascar     ..................................-T...-..............          462
Adome     ..................................-T...-..............          468
Amont     ..................................-T...-..............          467
Asiam     .......................................-..............          467
Abras     .T.....T..........................-T...-..............          463
Aotae     .......................................T..............          456
```

Strain details: Bio4, *Apiotrichum terrigenum* sp. nov. Bio4^T (FM212441); Ascar, *Apiotrichum scarabaeorum* CBS 5601^T (AB164372); Adome, *Apiotrichum domesticum* CBS 8280^T (AF444414); Amont, *Apiotrichum montevideense* CBS 6721^T (AF444422); Asiam, *Apiotrichum siamense* JCM 12478^T (AB164370); Abras, *Apiotrichum brassicae* CBS 6382^T (AF444436); Aotae, *Apiotrichum otae* JCM 12593^T (AB180196). Sequence accession numbers are shown in parentheses.

Note: For comparative purposes, the ITS region of *A. scarabaeorum* NCYC 3734 was also determined in this study and compared to the type strain (CBS 5601^T). Despite the fact the two *A. scarabaeorum* strains were isolated from very different substrates (CBS 5601^T, gut of larval scarab beetle; NCYC 3734, biogas reactor sample) and from different geographic locations (CBS 5601^T, South Africa; NCYC 3734, UK), both were found to have identical ITS sequences.

Table S1. Key growth characteristics for distinguishing between *Apiotrichum terrigenum* sp. nov. and *Apiotrichum scarabaeorum* strains

**Test:**	***A. terrigenum* sp. nov.**					***A. scarabaeorum****
	(UK)			(Norway)	(Hungary)	
	NCYC 3540	NCYC 3737	NCYC 3767	NCAIM Y.02175	NCAIM Y.02176	
Sucrose	+	+	s	-	-	+/s
Galactose	-	-	s	s	-	w/-
Sol. Starch	-	-	-	s	s	d/-
D-Xylose	-	-	-	-	-	+
Glycerol	s	s	-	+	+	+
Citrate	-	s	-	s	s	-

* Based on the combined results of strains CBS 4375 (cow rumen, New Zealand), CBS 5601^{T} (gut of larval scarab beetle, South Africa) and NCYC 3734 (biogas reactor, UK) (this study; Sugita, 2011).

Growth test results: +, positive; s, slow; d, delayed (latent); w, weak; -, negative.
Strain origins: Biogas reactor, NCYC 3540 & NCYC 3737; Soil, NCYC 3767 & NCAIM Y.02175; Sewage, NCAIM Y.02176
